# Tumour endothelial cell reprogramming orchestrates angiocrine signalling to drive chemoresistance in breast cancer

**DOI:** 10.1007/s10456-026-10063-7

**Published:** 2026-06-22

**Authors:** Jesus Gomez-Escudero, Eleni Maniati, Julie Holdsworth, Gordon Beattie, Maruan Hijazi, Matt Guelbert, Samar Elorbany, Pedro Cutillas, Jun Wang, Kairbaan Hodivala-Dilke, Gabriela D’Amico

**Affiliations:** 1 Barts Cancer Institute, Queen Mary University of London, Charterhouse Square, London, UK; 2 Biochemistry and Molecular Biology Department, Salamanca University, Salamanca, Spain; 3 UCL Cancer Institute, CRUK City of London Centre Single Cell Genomics Facility, University College London, London, UK; 4 Bioinformatics Hub, UCL Cancer Institute, University College London, London, UK; 5 Division of Cancer Biology, The Institute of Cancer Research, London, UK

**Keywords:** Angiocrine, Chemoresistance, Breast cancer, NF-κB, Doxorubicin

## Abstract

Despite its established role in breast cancer treatment, Doxorubicin treatment remains subject to adaptive resistance mechanisms that extend beyond cancer cell intrinsic alterations ultimately reducing therapy efficacy. Our study in a MMTV-PyMT-driven mouse breast cancer model reveals that prolonged Doxorubicin (Dox) exposure triggers significant reprogramming of the tumour vasculature, substantially altering the angiocrine landscape and shaping treatment outcomes. Notably, tumours that initially respond, but later revert, display an endothelial cell subclustering with activation of proliferative and NF-κB-dependent cytokine pathways. We further identify a novel endothelial subpopulation characterised by higher expression of drug clearance and oxidative metabolism markers, suggesting an active role in mitigating Dox efficacy and angiogenesis promotion. These findings substantiate endothelial plasticity as a critical mediator of therapeutic failure. By uncovering these vascular adaptations, our work provides a new perspective on the underlying mechanisms of Dox resistance and the prolonged efficacy of chemotherapy in breast cancer.

**Supplementary Information:**

The online version contains supplementary material available at 10.1007/s10456-026-10063-7.

## Introduction

Although substantial advances in early detection and therapeutic intervention have been made, breast cancer continues to be a major clinical challenge. Doxorubicin, a key component of breast cancer chemotherapy, is widely used for its potent antitumor activity; however, its prolonged efficacy is compromised by the development of chemoresistance and off-target toxicities [1, 2]. While extensive research has focused on cancer cell-intrinsic mechanisms of resistance [3–6], emerging evidence underscores the pivotal role of the tumour microenvironment, particularly the vascular compartment, in modulating therapeutic responses [6–11].

Endothelial cells (ECs) not only regulate drug delivery to tumour sites but also actively contribute to tumour progression through the secretion of angiocrine factors that influence cell proliferation, survival, and immune modulation [12–16]. Recent studies have highlighted that Doxorubicin (Dox) can induce profound functional alterations in ECs in other vascular beds, suggesting that the heterogeneity within the tumour vasculature may critically impact the overall chemotherapeutic outcome [13, 17–19]. Notably, the NF-κB signalling pathway, a key regulator of inflammation and cellular stress responses, has been implicated in mediating changes in angiocrine signals that can either support or counteract the efficacy of Dox [7, 20, 21].

In this study, we used a MMTV-PyMT (Mouse Mammary Tumour Virus–Polyoma Middle T antigen) transgenic mouse model of breast cancer to systematically dissect the effects of prolonged Dox treatment on EC dynamics. This widely used model provides a robust, reproducible, and clinically relevant system for investigating the biology of breast cancer, evaluating therapeutic strategies, and understanding the molecular mechanisms underlying tumour progression [22–24]. By integrating bulk and single-cell transcriptomic analyses, we reveal distinct EC subpopulations with dissimilar responses to Dox. Our findings not only delineate the heterogeneity of EC responses but also demonstrate how alterations in NF-κB-dependent angiocrine signalling contribute to the development of partial drug resistance. These insights advance our understanding of the interplay between the tumour vasculature and chemoresistance, and they may open new avenues for targeting the tumour microenvironment to improve therapeutic outcomes in breast cancer.

## Results

### Prolonged treatment with Doxorubicin leads to various alterations in endothelial cells that are associated with a different response pattern to the therapy

**Fig. 1:**
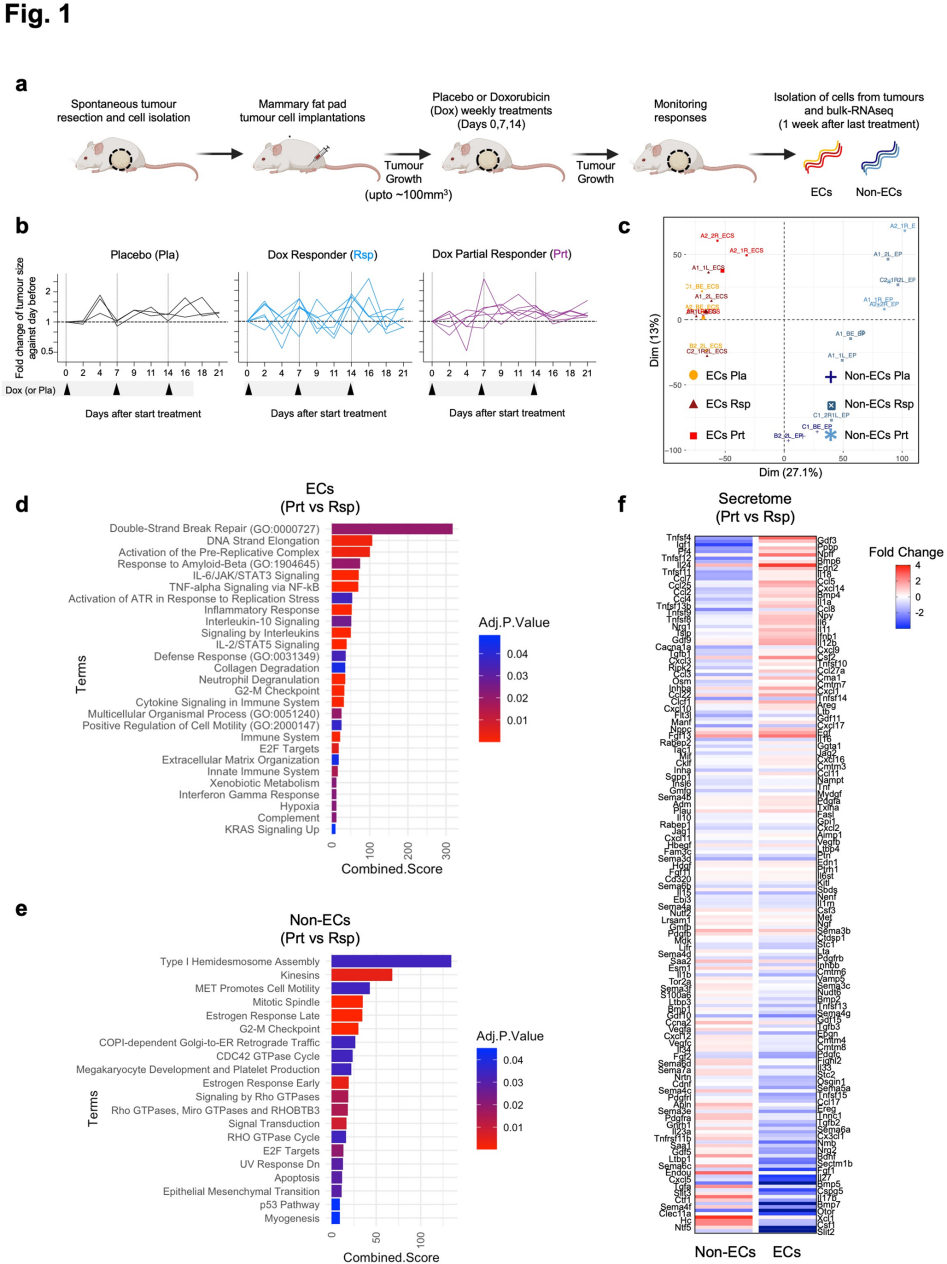
Prolonged Doxorubicin treatment promotes distinct response patterns in the MMTV-PyMT breast cancer model. (**a**) Cartoon showing the prolonged Doxorubicin treatment model (created with BioRender (BS294BTN5B). Wildtype (FVB/N) females were implanted with 10^5 cancer cells isolated from treatment naïve MMTV-PyMT (FVB/N) tumour bearing mice. When tumours reached approximately 100 mm³ in size, they were treated weekly, intraperitoneally, with 4 mg/kg of Doxorubicin (Dox) for three weeks, while size was monitored throughout the whole experiment. **(b)** Plots depict tumour size related to the previous measure size (as fold change). Placebo (Pla, *n* = 3 mice), Responders (Rsp, *n* = 6 mice), and Partial Responders (Prt, *n* = 6 mice). **(c)** Principal Component Analysis graph showing tumour endothelial cells (ECs) and tumour cancer cells (Non-ECs) bulk-RNA sequencing samples; prepared from Pla, Rsp and Prt to Dox treatment mice (*n* = 20 cell preparations in total; *n* = 3 for EC Pla, *n* = 2 for Non-EC Pla, *n* = 4 for EC Rsp, *n* = 5 for Non-EC Rsp, *n* = 3 for EC Prt, and *n* = 3 for Non-EC Prt). Bar plot shows top 20 pathway terms significantly deregulated in **(d)** ECs and **(e)** in Non-ECs, Prt versus Rsp comparisons. Colour gradient of bars represents adjusted p-values (Fisher’s exact test with Benjamini-Hochberg post hoc test for multiple hypotheses). **(f)** Heatmap shows differential expression of various secreted molecules (secretome) from Non-ECs and ECs isolated from tumours from Prt and Rsp to Dox-treated mice, Prt versus Rsp comparisons (DE as fold change, from normalized counts transformed using voom)

We utilised the MMTV-PyMT (Mouse Mammary Tumour virus-polyoma middle tumour-antigen) [22–24] transgenic mouse model of breast cancer to examine the impact of tumour ECs on Dox sensitivity and development of chemoresistance. We implanted cancer cells isolated from MMTV-PyMT-tumours into wildtype (MMTV-PyMT-negative) FVB/N females to ensure consistent tumour growth (Fig. 1a). To evaluate the effectiveness of prolonged Dox treatment (4 mg/kg dose, one time per week, over three weeks), we compared daily measurements of tumour size (Fig. 1b; Fig. S1a). In contrast, to the placebo control group (Pla), the Dox-treated group exhibited two distinct tumour growth response patterns. Some tumours showed frequent increases and sharp decreases in tumour size (fold change) throughout the treatment schedule, which we classified as Responders (Rsp) to prolonged Dox treatment. However, others showed less variation in tumour size fold change with fewer growth reductions, and we categorised these as Partial Responders (Prt) to prolonged Dox treatment. Our analysis further revealed that the Rsp group had a higher percentage of days with size reduction compared to the Prt group (Fig. S1b). Additionally, while both groups displayed similar tumour growth rates at the beginning of treatment, the growth rate for the Prt group increased significantly in the later stages of treatment suggesting a partial response to Dox. In contrast, the Rsp group did not acquire this increase in growth rate at later stages of treatment suggesting a more robust response to Dox therapy (Fig. 1b; Fig. S1a-b). Similar tumour growth patterns were observed in additional independent experiments (Fig. S1c-d).

To elucidate the factors influencing these differential responses to Dox, we examined transcriptomic changes in tumour ECs, which serve as barriers to drug delivery and are implicated in therapeutic resistance [7, 13, 19]. ECs were isolated from tumour cell suspensions, prepared from tumours that were harvested one week after last treatment, using magnetic antibody cell separation (MACS) combined with CD31 and ICAM2 antibodies specific for ECs (Fig. 1a-b). The unbound fraction of cells was enriched in epithelial cancer cells (Non-ECs), as reported previously [25]. 

Total mRNA from tumour ECs and Non-ECs preparations was subjected to bulk-RNA sequencing (Fig. 1a). Analysis revealed distinct enrichment of endothelial markers in tumour ECs, while Non-ECs exhibited increased epithelial markers (Fig. S1e). Principal Component Analysis (PCA) showed a clear differentiation between tumour EC and Non-EC samples, with Dox-treated tumour ECs clustering separately, suggesting distinct regulatory mechanisms (Fig. 1c). To gain insight into the distinct changes promoted by prolonged Dox in tumour ECs, we performed differential expression gene analysis by comparing Prt and Rsp, in both Non-ECs and tumour EC samples (Fig. S2a). Distinct regulatory patterns in tumour ECs were observed between the Prt and Rsp groups. The proportions of upregulated and downregulated unique genes differed compared with Non-EC (Fig. S2b). Compared with placebo, the EC Prt group showed less deregulated unique genes than the Rsp group (Fig. S2c), thus highlighting distinct regulatory responses between the two Dox-treatment groups.

EnrichR analysis [26–28] showed significant and distinct pathway changes in ECs and Non-ECs between Prt and Rsp to Dox groups (Fig. 1d, e). Tumour ECs had alterations in NF-kB transcription, secretome production, and cytokine signalling pathways (Fig. 1d). Compared with placebo, Rsp ECs mainly showed differences in replication and cell division pathways (Fig. S1f), whereas Prt ECs showed changes in NF-kB signalling, the secretome, and extracellular matrix pathways (Fig. S1g). Furthermore, differential expression of various secreted molecules was identified between Prt and Rsp tumour ECs (Fig. 1f). Collectively, our data suggests that Dox treatment reshapes EC transcriptional states in a manner that associates with divergent tumour responses to Dox therapy.

### Identification of distinct endothelial cell populations in prolonged Doxorubicin treated breast tumours

**Fig. 2:**
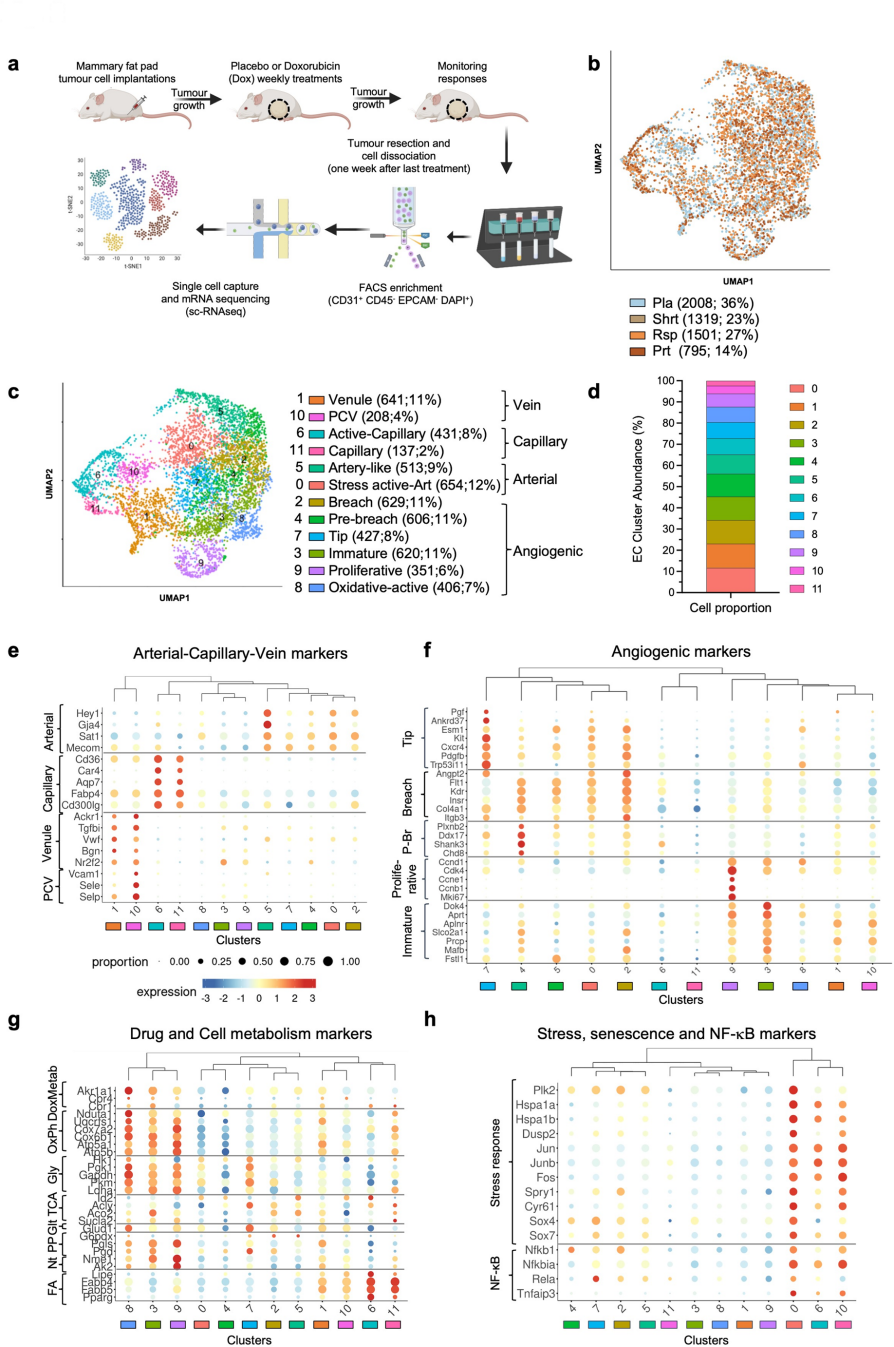
Fig. 2. Distinct endothelial cell populations detected by sc-RNAseq in Doxorubicin treated MMTV-PyMT tumour bearing mice. (**a**) Experimental design for the single-cell RNA sequencing (sc-RNA-seq) approach. Created with BioRender (MI294B6DD9). (**b**) UMAP visualises single endothelial cells (EC) isolated from MMTV-PyMT tumours from Placebo and Doxorubicin (Dox)-treated mice, after filtering low quality and all non-vascular endothelial cells (n = 3 pooled tumour samples per condition). Numbers in parenthesis represent cell counts. (**c**) Unsupervised clustering of distinct single cell EC populations. Numbers in parenthesis represent cell counts. (**d**) Percentage of cells in each EC cluster. Bubble plots for (**e**) arterial, capillary and vein (**f**) angiogenic, (**g**) drugs and cell metabolism, and (**h**). stress response and NF-kB pathway markers expressed by each EC cluster. Bubble size represents the percentage of cells in the cluster expressing a particular marker, and bubble colour represents marker expression levels (generated with ShinyCell app[91] based on normalized expression values)

To investigate the effects of prolonged Dox treatment on tumour EC dynamics at single-cell resolution, we carried out single-cell RNA sequencing (sc-RNAseq). For this, tumour EC enrichment was achieved by combining MACS and fluorescence-activated cell sorting (FACS) for CD31 + CD45- viable cells (Fig. 2a). Transcriptomic analysis excluded cells lacking CD31/Pecam1 and Cdh5 and verified the absence of markers for other cell types, including lymphatic (Lyve1), epithelial (Cdh1, Ktr5), fibroblast (Acta2), pericyte (Pdgfrb), and immune (Ptprc) cells (Fig. S3a-b). Unsupervised cluster analysis identified twelve distinct tumour EC populations (Fig. 2b-c), represented across all samples (Fig. 2d). The typical arterial-capillary-vein axis was assessed via specific markers for arterial (Gja4, Hey1), capillary (Crxc4, Cd36), and vein (Vwf, Ackr1), revealing populations with arterial characteristics in clusters 5 and 0, capillary markers in clusters 6 and 11, and venous markers in clusters 1 and 10, with cluster 10 classified as postcapillary veins (PCV) due to the additional expression of Selp and Vcam1 (Fig. 2e).

Next, we sought to identify characteristic markers that signify the angiogenic properties of tumour ECs as described previously [17, 29–32](Fig. 2f). Cxcr4, Trp53i11, and Pdgfb were notably enriched and detected in cluster 7 (Fig. 2f). Additionally, pathways related to migration, chemotaxis, and extracellular matrix remodelling were significantly represented in this cluster (Fig. S4a), further confirming the identity of these cells as “Tip” ECs [31, 32]. Interestingly, some markers associated with this population were also found in another cluster, which we referred as cluster 2, albeit at much lower levels. Literature review indicates that this cluster displays genes typical of a specific angiogenic population known as “Breach” ECs [32]. Furthermore, cluster 4 expresses markers indicative of a precursory state of these “Breach” ECs and we therefore classified it as “Pre-Breach” (P-Br) ECs (Fig. 2f). These cells have only been identified in tumours and are believed to assist “Tip” cells in spreading due to their podosome-like characteristics [32]. Consequently, pathways related to migration, chemotaxis, and extracellular matrix remodelling were also significantly represented in this cluster, similar to what was observed in cluster 7 (Fig. S4a).

We then examined proliferative cells by assessing Ki67 expression, alongside with cyclins and Cdks expression, revealing that cluster 9 comprises “Proliferative” ECs (Fig. 2f). Notably, Cdk4 was also expressed in cluster 3, indicating these cells may be in the cell cycle stage. This cluster is characterised by an enrichment of immature markers, suggesting it represents cells that are transitioning into or out of the cell cycle, thus we have designated them as “Immature” ECs (Fig. 2f). Pathway analysis further demonstrated that cluster 9 exhibits a significant upregulation of cell cycle and mitotic pathways (Fig. S4a).

Gene expression related to nucleotide metabolism, oxidative phosphorylation, fatty acid oxidation, and glycolysis across clusters (Fig. 2g) were identified, revealing that both the “Proliferative” (cluster 9) and “Immature” (cluster 3) cells are metabolically active, exhibiting high levels of glycolytic and oxidative phosphorylation pathways to fulfil their energy demands. “Tip” cells (cluster 7) are characterised by the expression of glycolytic enzymes that facilitate migration, corroborating previous findings [33]. Fatty acid oxidation is essential for the migration and survival of active, capillary proliferating EC (“stalk” cells) during angiogenesis. It also allows mature capillary EC (“phalanx” cells) to maintain a non-proliferative, quiescent state and support vascular integrity and adaptation to metabolic stress [34–36]. This metabolism was found to be restricted to capillary clusters 6 and 11. Interestingly, we discovered a subpopulation of ECs that has not been described in the literature. This cluster (Cluster 8, “Oxidative-active”) exhibited similarities with “Immature” and “Proliferative” cells, characterised by increased energetic metabolic pathways and a lack of specific angiogenic markers, while also expressing the highest levels of Dox clearance markers (Akr1a1, Cbr4). Furthermore, analysis of Hallmark pathways indicated possible involvement in hypoxic regions and of glycolysis pathways (Fig. S4b), reported features of an aggressive tumour cell phenotype [37].

Dox treatment and limited nutrient availability create stress in ECs that they must overcome for survival. Our analysis identified three clusters enriched in genes linked to EC stress response and activation: Hsp1a1, Plk2, Junb, Sox4, and Cyr61 (Fig. 2h). These include cluster 0, cluster 6, and cluster 10. Cluster 6 consists of active capillary ECs, while cluster 10 is characterised by postcapillary veins. Cluster 0, which shares markers with arterial cells (Stress-active-Art) (Fig. 2e), shows limited metabolic activity (Fig. 2g). Notably, genes associated with the NF-κB pathway, such as RELA and NFKBIA, are enriched in these clusters (Fig. 2h), with Hallmark pathways analyses revealing significant enrichment for the TNF/NF-κB pathway (Fig. S5). Overall, these findings highlight the presence of a highly heterogenous EC landscape in prolonged Dox treated tumours.

### NF-kB pathway activation influences angiocrine signals in tumour endothelial cells from prolonged Doxorubicin treated mice

**Fig. 3:**
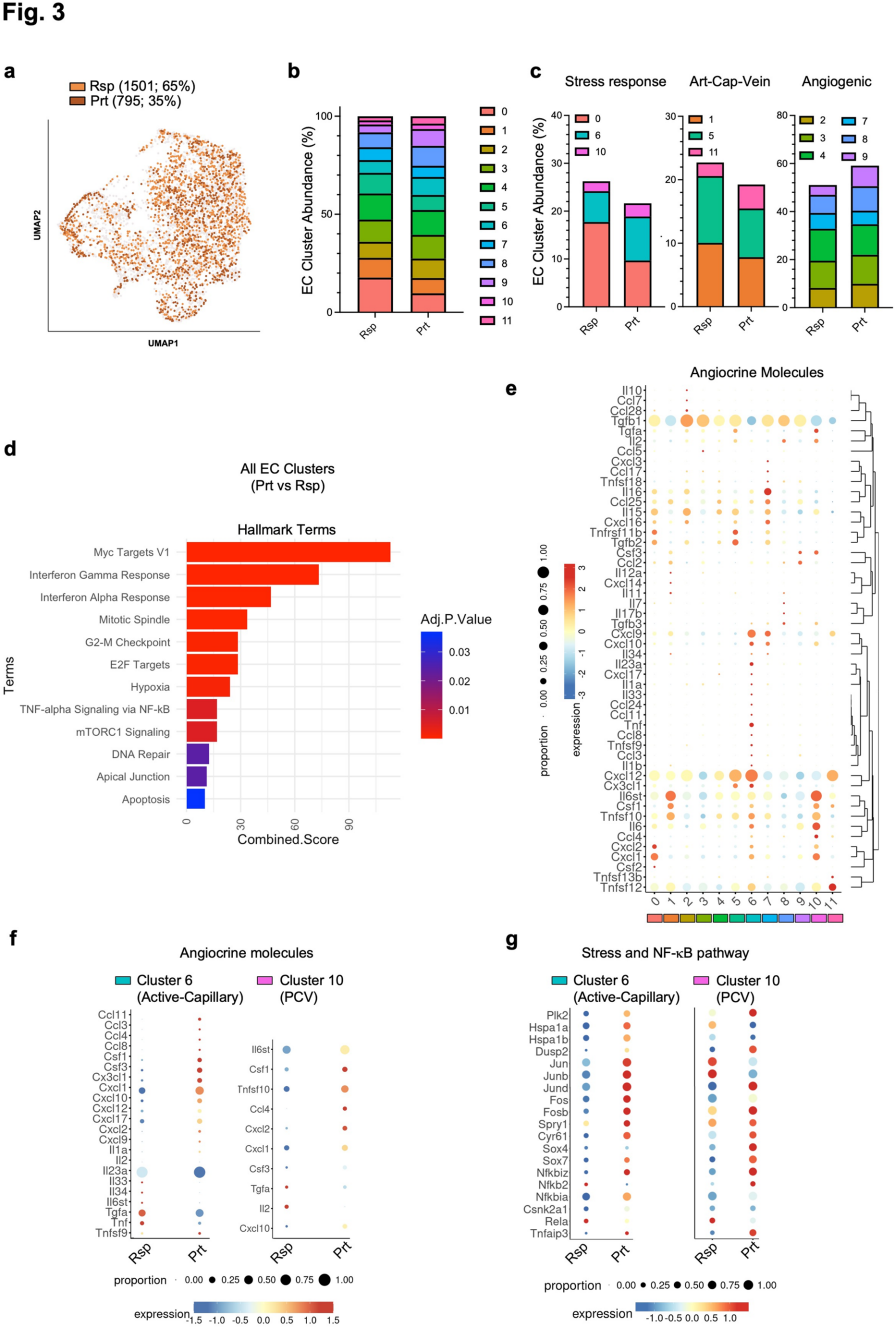
Angiocrine factors alterations in specific endothelial cells populations associates with response to prolonged Doxorubicin treatment. **a**. UMAP visualises single endothelial cells (ECs) isolated from MMTV-PyMT tumours from Responders (Rsp), and Partial Responders (Prt) prolonged Doxorubicin (Dox)-treated mice. (n = 3 pooled tumour samples per condition). Numbers in parenthesis represent cell counts. **b**. Cell cluster abundance in percentage, in each Dox treatment group. **c**. Abundance of clusters (in percentage) in each indicated EC cluster category, in each Dox-treated group. **d**. Significantly deregulated Hallmark pathway terms in Prt versus Rsp ECs. Colour gradient scale represents adjusted p-values (Fisher exact’s p-value). **e**. Bubble plot shows representative angiocrine molecules expression within each cluster in Rsp versus Prt ECs. **f**. Angiocrine molecules, and **g**. Stress response and NF-kB pathway molecules from angiocrine expression within clusters 6 and 10, in Rsp versus Prt ECs. Bubble size represents the percentage of cells in the cluster expressing a particular marker, and bubble colour represents marker expression levels. Plots generated with ShinyCell app[91] from normalized gene expression data.

We conducted a comparative analysis of the abundance of cellular subpopulations in prolonged Rsp and Prt Dox-treated samples (Fig. 3a-b). Notably, angiogenic cell clusters accounted for 50–60% of total cells in both Dox-response samples, reflecting the high angiogenic characteristics of breast tumours (Fig. 3b-c). Multiple studies support that cancer therapy, including chemotherapy can promote angiogenesis, tumour regrowth, and progression after therapy [38–42]. We observed a decrease in Rsp ECs compared with partial Prt ECs, attributed to a higher proportion of proliferative cells (cluster 9) and oxidative-active cells (cluster 8) in the Prt group. Additionally, there was a significant reduction in the ratio of larger vessels versus small vessels, with a higher capillary (clusters 6 and 11) and postcapillary venule (PCV, cluster 10) proportions (Fig. 3b-c). Hallmark terms pathway analysis revealed that cell cycle regulation, interferon responses, Myc activation, hypoxia response, and NF-kB signalling were among the top pathways differentially regulated between Prt and Rsp across all tumour EC clusters (Fig. 3d). Given that our bulk-RNAseq showed a deregulation of the NF-kB pathway (from Fig. 1d) and differences in the expression of angiocrine signals between Prt and Rsp ECs (from Fig. 1f), we aimed to investigate the expression of angiocrine molecules to determine which clusters of ECs produce them.

Certain angiocrine molecules, such as Cxcl12, Il6st, Tgfb1, Tnfsf10, and Tnfsf12 were widely expressed, although their levels varied across different clusters (Fig. 3e). Notably, activated capillaries and postcapillary vein ECs (clusters 6 and 10) produced the highest levels of angiocrine molecules and were primary sources of various chemokines and cytokines involved in immunomodulation, including Il6, Il6st, Cxcl9, Cxcl10, Tnf, Csf1, Csf3, Cxcl12, Cxcl1, and Cx3cl1. They also presented the greatest number of significant pathway terms related to cytokines and interleukins in the gene and Hallmark terms analysis (Fig. 3e; Fig. S6; Fig. S7). Comparison between the two response groups to Dox, showed higher expression of angiocrine molecules in Prt ECs (Fig. 3f). Moreover, the increased expression of these genes in Prt ECs corresponds with pathways for stress and NF-κB (Fig. 3g), suggesting that resistance to prolonged Dox treatment may be linked to higher angiocrine expression molecules in clusters 6 and 10 due to NF-kB pathway activation.

To investigate the initial changes in angiocrine signals, we next analysed the acute Dox treatment (hereinafter referred to as Shrt), which reflects the first week of the prolonged treatment, where tumour growth rates were similar between the Rsp and Prt groups (Fig. 1b). All previously described EC populations were represented across samples (Fig. S8a-b) with about half identified as angiogenic (Fig. S8c). After Shrt Dox treatment, we observed a reduction in immature and proliferative ECs (clusters 3 and 9) and a slight increase in oxidative ECs (cluster 8) (Fig. S8c). Myc targets showed significant differences in the Shrt EC clusters, possibly accounting for the reduction in proliferative ECs (cluster 9) (Fig. S8d). In contrast to prolonged Dox treatment, the NF-kB pathway did not show significant changes, although clusters 6 and 10 show variation in some stress and NF-kB pathway gene expression (Fig. S8e). Characteristic angiocrine molecules in these clusters also exhibited varied regulation levels (Fig. S8f).

### Doxorubicin-driven angiocrine signals influence the response of breast cancer cells to treatment in vitro

**Fig. 4:**
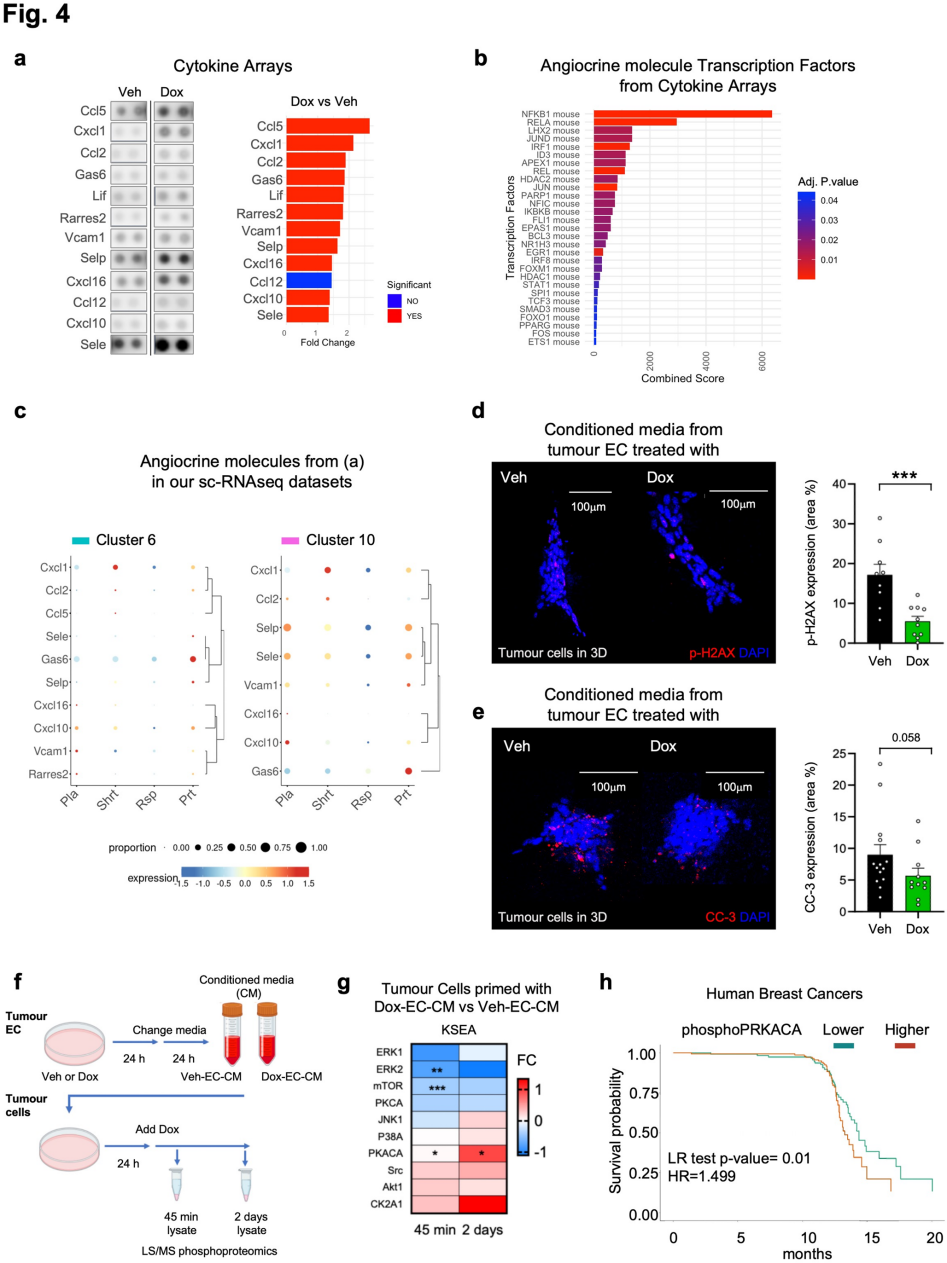
Doxorubicin-driven endothelial angiocrine signals promote molecular changes in tumour cancer cells related with development of resistance to Dox-treatment. **a**. Cytokine arrays analysis of endothelial cells (ECs) isolated from MMTV-PyMT tumours and treated in culture with PBS vehicle (Veh) or 0.125µM Doxorubicin (Dox) for 24 h. Selected cytokines and quantification bar plot are shown. n = 4 replicates; NO = not statistically significant; YES = p < 0.05 (two-sided Student’s t-test). (**b**). Bar plot of Trrust transcriptional factors using the significantly deregulated cytokines from the arrays in (**a**). Colour gradient of bars represents adjusted p-values (Fisher exact’s p-value). (**c**) Bubble plot shows expression of significant deregulated genes from arrays in (**a**) assessed in the angiocrine clusters 6 and 10 from the sc-RNAseq analysis. Genes were cluster according to their expression across the samples. Colour gradient of bars represents adjusted p-values (Fisher exact’s p-value). Representative images from MMTV-PyMT tumour-derived cancer cell spheroids pre-treated with conditioned media from ECs treated in culture with PBS vehicle (Veh) or Doxorubicin (Dox) for 24 h, cancer cells were next treated with Veh or 0.125µM Dox for further 48 h, and last stained for (**d**) p-H2AX or (**e**) Cleaved Caspase-3 (CC-3). Scales, 100 μm. Quantification plots of p-H2AX area or CC-3 area per DAPI-positive area are shown. ****p < 0.0001 (two-sided Student’s t-test). (**f**). LS/MS phosphoproteomics experimental design for MMTV-PyMT tumours-derived cancer cells sample analysis. Created with BioRender (CW294BUSKT). (**g**). Kinase Significant Enrichment Assay (KSEA) analysis. FC, fold change. n = 4 cell lysates for each condition; *P < 0.05, **P < 0.01, ***P < 0.001 (two-sided Student’s t-test). (**h**) Kaplan-Meier plot shows disease progression/survival in high and low phospho-PRKACA expression human breast cancer patient groups from LinkedOmics database [48]. Cox proportional hazard ratios, with the rank P value is shown

To study the impact of angiocrine signals from ECs in response to Dox on tumour cancer cells behaviour in vitro, we used Dox at 0.125µM dose. This dose was chosen based on that (1) isolated tumour cancer cells from treatment-naïve MMTV-PyMT tumours (Non-EC fractions) showed approximately a 50% reduction in survival rate when assessed by crystal violet assays (Fig. S9a), (2) previous work from our laboratory [7] shown that 0.125µM Dox treatment of endothelial cell cultures generates cytokine enriched conditioned media, thus a relevant feature for our current study; and (3) is similar to that used in the in the literature [7, 43, 44]. Isolated ECs from treatment-naïve MMTV-PyMT tumours (EC fractions), were treated with 0.125µM Dox for 24 h and then subjected to cytokine arrays analysis. Dox treatment of ECs resulted in the upregulation of several angiocrine molecules, including Ccl5, Ccl2, Cxcl10, Cxcl16, Cxcl1, and Selp (Fig. 4a, Fig. S9b-d). Notably, we found that NF-kB is a major regulator of these significantly regulated molecules (Fig. 4b) and that bulk-RNAseq results further support the increased expression of these molecules (Fig. S9e). Moreover, these angiocrine molecules were predominantly upregulated in clusters 6 and 10 in our sc-RNAseq analysis, in the Prt ECs when compared to Rsp ECs (Fig. 4c).

Tumour spheroid cultures provide a three-dimensional model that closely mimics the architecture and microenvironment of in vivo tumours, making them valuable for evaluating anticancer drug responses [45–47]. To analyse the effect of Dox-driven angiocrine signals on cancer cells, treatment-naïve MMTV-PyMT tumour-derived cancer cells, were used to form spheroids, and subsequently were treated with conditioned media from ECs pre-treated with Dox and subsequently washed (Fig. 4d-e). In comparison to conditioned media (CM) from PBS control (Veh)-treated ECs, CM from Dox-treated ECs protected cancer cells from DNA damage and death, indicated by a reduction in p-H2AX and active Caspase-3 levels respectively (Fig. 4d-e; Fig. S10a-b). Further, crystal violet survival assays performed in MMTV-PyMT tumour-derived cancer cell in two-dimensional cultures, subjected to same CM and Dox treatments above described, shown that CM from Dox-treated ECs protected Dox-treated-, but not Veh-treated-, cancer cells from death (Fig. S11a).

Mass Spectrometry phosphoproteomic analysis was performed to decipher changes occurring in the tumour cells after the treatment with the EC CM, which may protect them from Dox damage, and promote the resistance to the treatment. Analysis showed that CM from EC treated with Dox promoted significant reduction of ERK2 and mTOR and, in contrast, PRKACA was found significantly upregulated (Fig. 4f-g). Taken together these changes in signalling pathways may lead to the functional changes observed in the tumour cancer cells as we observed significant regulated pathways related with DNA damage repair from the phosphoproteomic data (Fig. S11b). Furthermore, to test whether our results could extend to human disease, we interrogated human breast cancer patient phosphoproteomic datasets [49], we found that high phospho-PRKACA expression levels in breast cancer patients associates with poor survival outcome (Fig. 4h). Overall findings not only delineate the heterogeneity of EC responses but also demonstrate how alterations in NF-κB/PRKACA dependent angiocrine signalling contribute to the development of partial resistance to Dox.

## Discussion

ECs are increasingly recognised as active regulators of tumour progression rather than passive conduits for nutrient delivery. Beyond their vascular functions, ECs modulate senescence, metastatic niche formation, immune responses, and therapeutic resistance through dynamic angiocrine signalling [13, 14, 50, 51]. Here, we investigated how endothelial heterogeneity and angiocrine programmes influence sensitivity to Dox, a first line DNA-damaging agent in breast cancer therapy [1, 2].

Using the MMTV-PyMT mammary carcinoma model with a tumour cell implantation approach, to standardise experimental timing, we observed heterogeneous tumour responses to prolonged Dox treatment. Some tumours displayed repeated strong growth regression, whereas others showed only transient responses followed by increased regrowth, suggesting partial resistance. Similar adaptive resistance has been described in BRCA1-deficient breast cancer models treated with DNA-damaging agents and in other malignancies where resistance arises through tumour microenvironmental remodelling, immune suppression, or malignant cell plasticity [52–54].

Bulk transcriptomic profiling revealed pronounced tumour microenvironment compartment-specific differences. ECs underwent substantial transcriptional reprogramming associated with partial or “failed” responses to Dox, whereas epithelial cancer cell-enriched populations remained relatively homogeneous. These findings align with previous reports showing that chemotherapy profoundly alters EC transcriptional states, promoting angiogenic activity, impairing vascular differentiation, and inducing senescence or extracellular matrix remodelling programmes [55–58]. Notably, angiocrine pathways were selectively altered in ECs from partially Dox-responsive tumours, underscoring endothelial-specific mechanisms of resistance.

Chemotherapy-induced modulation of the endothelial secretome has emerged as a key determinant of tumour progression and therapeutic failure. EC-derived angiocrine factors can establish chemoresistant niches, as shown by Serpine1 secretion in breast cancer lung metastases and IL-8-mediated drug resistance [59, 60]. Angiocrine signalling also reshapes extracellular matrix organisation, cell adhesion, and angiogenesis, collectively diminishing tumour sensitivity to cytotoxic therapies [61]. Our findings extend these observations by demonstrating that Dox similarly reprograms EC secretory profiles in mammary breast cancer tumours.

Single-cell RNA sequencing revealed extensive vascular heterogeneity, including arterial, venous, capillary, and angiogenic EC populations corresponding to previously described tip and proliferative states. Multiple studies support that cancer therapy, including chemotherapy, can induce host responses, such as activation of endothelial progenitor cells or mobilisation of bone marrow–derived cells, which paradoxically promote angiogenesis, tumour regrowth, and progression after therapy [38–42]. These subsets likely contribute to abnormal, leaky tumour vasculature, impairing drug delivery, promoting hypoxia, and fostering resistance [62–64]. Restoration of vascular maturity has been shown to improve chemotherapy efficacy, supporting a causal role for endothelial dysfunction in therapeutic resistance [64].

Among these populations, we identified a distinct “Oxidative-active EC” subset characterised by elevated oxidative phosphorylation markers and cyclin D1 expression, consistent with a G1 cell-cycle state [65]. These cells exhibited enhanced glycolysis, oxidative phosphorylation, glutamine metabolism, and drug metabolism without expressing canonical markers of immaturity. While hypoxia-associated gene expression was present, it was not markedly elevated relative to other EC subsets. Oxidative EC metabolism has been implicated in pathological angiogenesis, tumour growth, and chemoresistance [66–68]. We propose that this population contributes to Dox resistance by sustaining dysfunctional vasculature, limiting drug delivery, and releasing survival-promoting angiocrine factors.

Angiocrine signalling also plays a central role in shaping tumour-immune interactions. Our single-cell analyses revealed distinct angiocrine expression profiles across EC subsets, with active capillary and postcapillary venous ECs showing enrichment of molecules linked to therapy response and immune regulation. Capillary ECs are specialised for molecular exchange and secretion, supporting tumour cell stemness and stress adaptation, while postcapillary venules secrete factors involved in immune cell recruitment and coagulation. In breast cancer, the spatial interplay between these compartments within angiogenic regions correlates with increased metastatic risk and poorer prognosis [50, 51]. Furthermore, postcapillary venules can be reprogrammed into high endothelial venules, generating immune-permissive niches during immunotherapy [69].

Angiocrine gene expression is regulated by multiple transcriptional programmes, including ETS family members, Notch signalling, hypoxia-inducible factors, ERG, AP-1, EphA2, and NF-κB [13, 15, 70–73]. NF-κB signalling is of relevance to chemoresistance, as Dox-induced oxidative stress activates this pathway, driving pro-inflammatory cytokine and pro-angiogenic factor expression [7, 74, 75]. Consistent with this, we observed elevated NF-κB pathway activity within angiocrine EC clusters, correlating with partial tumour responses and enrichment of these factors in EC-derived secretomes.

Functionally, conditioned media from Dox-treated ECs reduced tumour cell sensitivity to Dox-induced DNA damage and cell death. Our phosphoproteomic analyses revealed an initial suppression of mTOR and ERK signalling, that is, respectively sustained and further increased over time; and a pronounced activation of PRKACA. While mTOR and ERK signalling are established mediators of Dox resistance, emerging evidence suggests that dysregulated PRKACA activity promotes tumour survival [76]. Although much of the literature finds that ERK2 phosphorylation promotes chemoresistance, some evidence suggests that reducing ERK2 phosphorylation may also trigger Dox resistance mechanisms in various tumour cells, including breast cancer cells, potentially via dormancy or reduced cell death [77–79]. Additionally, inhibiting ERK activation may also increase DNA-damage repair and reduce p-H2AX [80]. Moreover, PKA is known to exert negative regulation of ERK2 activity [81, 82] and PKA activation in DNA damage-repair has also been reported in yeast [83–85]. We speculate that EC-derived angiocrine factors may dynamically affect ERK2, PKA and other kinases activity contributing to DNA-damaging chemotherapy resistance, reflected by the p-H2AX levels in cancer cells. Overall, our data suggest that angiocrine signals signpost chemoresistance through PRKACA activation, thus representing a potential therapeutic vulnerability.

Previous work has shown that DNA-damaging agents such as doxorubicin can affect EC-derived angiocrine factors [86]. For example, genetic ablation of EC-FAK, can affect the doxorubicin driven DNA-damage-induced angiocrine cytokine profiles, which in turn are associated with changes in doxorubicin effects on tumour cell survival and tumour growth. These effects correspond with p-H2AX Here we show that levels of chemoresistance are associated with altered angiocrine profiles, including IFN-γ, TGF-β, or IL-1β [87, 88], that have been reported to modulate ERK activation/inactivation, or PKA status [89]. Thus, the effects are an impairment of drug action, but the exact mechanisms remain unknown and will be the subject of a follow-on study.

In conclusion, our work reveals a distinct regulation of ECs within mammary tumours that correlates with the response to Dox. By dissecting EC heterogeneity, we identified specific subpopulations uniquely responsible for producing angiocrine signals that shape both therapeutic response and the vascular landscape of the tumour. Importantly, we demonstrate that the endothelial secretome actively modulates breast cancer cell sensitivity to Dox through the regulation of defined phosphoprotein signalling pathways. Together, these findings uncover a previously underappreciated role of EC in therapy response and highlight new opportunities to exploit the vascular niche for therapeutic intervention.

## Materials and methods

### Mice experiments and treatments

FBV/N background MMTV-PyMT-positive mice were used for this study. Health screens were performed in accordance with UK Home Office guidelines to monitor the health of rodent colonies, and to confirm free status of pathogens. Animals were housed in groups of 4–6 mice per cage in a 12 h light/dark cycle, with controlled room temperature (21 ± 1 °C) and relative humidity (40–60%). Animals had access to food and water *ad libitum*. To standardise growth of tumours and to perform treatments at the same size, we isolated epithelial malignant cells from tumours of MMTV-PyMT-positive mice, that were implanted in the fourth mammary fat pad of FBV/N Widtype females. 10^5^ cells were implanted per mice in a drop of 10 ul mixed 1:1 with Matrigel (Corning, 356234). Once the tumours reached around 100–200 mm^3^ of volume, mice were treated intraperitoneally with 4 mg/kg of Doxorubicin (Dox) or Placebo. For short Dox treatment, we treated the mice once, and 48 h after they were sacrificed, and tumours were processed. For the prolonged Dox treatment, mice were treated once weekly, for a period of 3 weeks with the same dose.

### Tumour digestion and cell isolation

Tumours were removed and cut to small pieces with a scissor. Then they were digested using 0.1% collagenase type I diluted in DMEM (Gibco), supplemented with 10% of EGM2 media (Promocell), 1% of BSA (Sigma), DNAse I, 100 mM HEPES and 2 mM EDTA. First the mix of tumour pieces was mixed with the digestion media, and tumours were processed in gentleMACS™ Dissociator (Miltenyi), then incubated at 37 degrees for 30 min, and then processed again in the Dissociator. Homogenized tumours were filtered with a 70-um filter and then the cell pellet was subjected to different procedures. ECs were enriched using a mix of antibodies against CD31, CD144 and ICAM2 (BD) and 30 ul of Dynabeads™ Sheep anti-Rat IgG (ThermoFisher). For bulk RNAseq, cell preparation was directly lysed to isolate mRNA. The non-binding fraction was used to enrich epithelial cells [25]. For single cell RNA-seq, endothelial cells were isolated from a pool of two mice per condition using cell death removal and EC isolation bead kits from Miltenyi. To obtain a pure population of ECs, we selected DAPI-, CD45-, EPCAM-, CD31 + cells by FACS. Cells were immediately processed for sc-RNAseq.

### Bulk-RNA sequencing and analysis

RNA was extracted using the Qiagen RNeasy kit (Cat no. / ID. 74104) according to the manufacturer’s instructions. Briefly, the cells were lysed with the provided Lysis Buffer, loaded into columns and centrifuged. Columns were washed several times with Wash Buffers (Cat no. / ID. 74104) and then eluted into an Eppendorf. Library prep and RNA sequencing were carried out by the Queen Mary University of London, Genome Centre Facilty, using ribosomal depletion. Sequencing was performed on the Illumina NextSeq platform yielding ~ 17 million reads, paired-end, 75 bp length. RNASeq samples were mapped to the mouse genome GRCm38 (mm10) using hisat2 (v2.1.0). Number of reads aligned to the exonic region of each gene were counted using htseq-count. Only genes that achieved at least one read count per million reads (cpm) in at least 25% of the samples were kept. Conditional quantile normalization was performed accounting for gene length and GC content, and a log2-transformed RPKM expression matrix was generated. Differential expression analysis was performed using R packages EdgeR and ‘limma’ R. Briefly, normalized counts were transformed using voom. A linear model with an intercept-free design was fit to the voom-transformed data, with empirical Bayes (eBayes) applied to stabilize variance estimates. Pairwise contrasts were defined between the experimental groups to identify differentially expressed genes across the relevant comparisons. Statistical significance was assessed using moderated t-tests, and p-values were adjusted for multiple testing using the Benjamini-Hochberg (BH) correction. A threshold of BH adjusted p-value < 0.05 was used to define significantly differentially expressed genes in the purity check of epithelial vs. endothelial cell samples (Fig S1e). For all other differential expression contrasts, a nominal p-value < 0.05 threshold was used to define significantly differentially expressed genes, due to sample heterogeneity within the treatment group contrasts. PCA plot was generated using R packages factoextra and FactoMineR. Heatmaps were generated using R package ComplexHeatmap.

### Processing of single-cell RNA sequencing

Single-cell suspensions were prepared and Live CD45- CD31 + populations were sorted. After this, cells were loaded and processed on the 10X Chromium Controller using the CG000331 Chromium Next GEM Single Cell 5’ Reagent Kits v2 (Dual Index) User guide enabled us to process the gene expression and endothelial profiling of the samples. Each library can be recognised by its unique sample index. Sample sequencing was performed at Novogene using a Novaseq 6000 platform and PE150 sequencing strategy. Gene expression reads were aligned using cellranger count (v6.0.1). Expression matrices were analysed using the Seurat package (v4.0.3). Starting from 2911 (Pla), 1834 (Shrt), 1882 (Rsp) and 1211 (Prt) cells per condition, the cells with mitochondrial reads making up > 10% of total read content or with less than 400 genes detected were removed. Multiple filtering steps were performed using DoubletFinder (v2.0.3) using author-recommended settings. Final number of 2008 (Pla), 1391 (Shrt), 1501 (Rsp) and 795 (Prt) cells per condition. Datasets were normalized using SCTransform (v0.3.2) using the top 3,000 variable genes. Principal component analysis (PCA) and uniform manifold approximation and projection (UMAP) dimensional reduction (dims = 1:30) was then performed using RunPCA and RunUMAP. Clustering was performed using FindClusters, using a resolution of 0.8. All differential gene expression analyses were performed on log normalized gene expression values (using NormalizeData, default parameters) using the MAST algorithm [90] within FindMarkers. To aid visualisation, sc-RNAseq data was packages into an interactive interface using ShinyCell [91]. Venn diagrams of deregulated genes were created with Venny app (https://bioinfogp.cnb.csic.es/tools/venny/index.htm).

### Gene ontology, terms and transcriptional factors analysis

Significant differential expressed genes were loaded into EnrichR [26–28] and Hallmark, Gene Ontology process or TF perturbations were analysed. Combined Score is described as c = log(p) * z, where c = the combined score, p = Fisher exact test p-value, and z = z-score for deviation from expected rank.

### Cytokine arrays

Endothelial cells isolated from treatment-naïve MMTV-PyMT tumours that were treated in vitro with PBS (vehicle, Veh) or Doxorubicin (0.125 µM) for 24 h and whole-cell lysates were extracted. Mouse cytokine arrays (Proteome Profiler ARY006, R&D Systems) were processed according to the manufacturer’s instructions using 100 µg of lysates (in 3% SDS, 60 mM sucrose, 65 mM Tris-HCl pH 6.8) per membrane. Pixel intensity analysis was used for quantification using Image J software (http://imagej.nih.gov/ij).

### In vitro doxorubicin treatment

Endothelial cells isolated from treatment-naïve MMTV-PyMT tumours were treated in vitro with PBS (vehicle, Veh) or Doxorubicin (0.125 µM) for 24 h. Media was changed and recovered after another additional 24 h. 1000 treatment-naïve MMTV-PyMT-tumour derived cancer cells were seeded in drops of 20 µl of 10%FBS DMEM containing 0.25% of methylcellulose (Sigma-Aldrich) and left overnight to form spheroids using the “hanging drop” method [33, 92, 93]. Briefly, drops where seeded using a multichannel pippete on the lid of a 15 cm dish, and then the lid was flipped over the dish (containing PBS). 24 h after incubation at 37 °C, drops were carefully collected with a P1000 tip, centrifuged, embedded in 4 mg/ml matrigel gels and incubated at 37 °C to solidify for 1 h. Conditioned media from ECs was added for 24 h to the gels, then spheroids were treated with PBS (vehicle, Veh) or Doxorubicin (0.125 µM) for extra 48 h. For the 2D experiments, 3000 cells were seeded and primed with conditional media from tumour ECs for 24 h and then treated with Doxorubicin for 72 h. Cells were fixed with cold methanol, and stained with crystal violet (diluted in 20% ethanol) for 15 min. After several washes with water, the plates were left to dry. Day after crystals were dissolved with methanol and quantify in a spectrophotometer at 590 nm.

### Immunostaining of spheroids

Gels were fixed in 1% PFA PBS for 30 min and washed in PBS. Gels were then permeabilized and blocked for 2 h in blocking and permeabilization (BP) buffer (2% BSA, 0.3% Triton X-100, 0.3 M glycine, 0.2% azide, and 1% goat serum in PBS). Gels were incubated with primary antibodies (anti-phosphohistone γH2AX (p-H2AX, Ser 139) antibody (Sigma-Aldrich, clone JBW30) or anti-cleaved caspase 3 (Cell Signalling, 9661) for overnight in the same buffer. The next day, gels were post-fixed in 4% PFA for 15 min. After a wash and 30 min in BP buffer, gels were incubated with appropriate secondary antibodies. Spheroids were imaged using an Laser Scanning Confocal Microscope (Zeiss 710) at 10x with NA 0.3 magnification, a 405 nm (DAPI) and 568 nm (TRITC) lasers were used. The area of positive staining of the proteins of interest was calculated within each spheroid and normalised to the area of the spheroid using Image J software (http://imagej.nih.gov/ij).

### Proteomic and phosphoproteomics analysis

Endothelial cells isolated from MMTV tumours that were treated with or without Doxorubicin (0.125 μm) for 24 h. Media was changed and recovered after another additional 24 h. Epithelial cells isolated from MMTV tumours, were treated with conditioned media from ECs for 24 h and then treated with 0.125 µM Doxorubicin for 45–48 h. Then cells were washed twice with ice cold PBS supplemented with phosphatase inhibitors (1 mM Na_3_VO_4_, 1 mM NaF). Cells were lysed in Urea Buffer [8 M Urea in 20 mM HEPES (pH 8.0)] supplemented with phosphatase inhibitors (1 mM Na_3_VO_4_, 1 mM NaF, 1 mM β-glycerol-phosphate, 2.5 mM Na_4_P_2_O_7_). Cell lysates were centrifuged for 10 min at 20,000 x *g* at 4 °C and supernatant transferred to fresh low protein binding tubes. Protein was diluted in a volume of 400 µl in Urea Buffer. Samples were sequentially incubated at 24 °C, shaking, with 10 mM dichlorodiphenyltrichloroethane (DDT) for 1 h and 16.6 mM iodoacetamide (IAM) for 30 min. Urea concentration was diluted to 2 M with 1.2 ml of 20 mM HEPES (pH 8.0), 90 µl of conditioned trypsin beads [50% slurry of TLCK-trypsin (20230; Thermo-Fisher Scientific Inc, Waltham, MA, USA) conditioned with 3 washes of 20 mM HEPES (pH 8.0)] were added, and samples were incubated overnight at 37 °C with shaking. Trypsin beads were removed by centrifugation at 2,000 x *g* for 5 min at 5 °C and 140 µg of digested protein was used for phosphoproteomics; 20 µg were used for proteomics. Samples were desalted using Carbon C18 Top tips (TT2MC18; Glygen, Thermo-Fisher Scientific Inc). CTop tips were activated with 100 µl of elution solution (70% acetonitrile (ACN), 0.1% trifluoroacetic acid (TFA)) and equilibrated twice with 200 µl of wash solution (1% ACN, 0.1% TFA). Samples were loaded and columns washed twice with 200 µl of wash solution (1% ACN, 0.1% TFA). For phosphoproteomics, phosphopeptides were eluted 4 times with 50 µl of glycolic acid solution 1 (1 M glycolic acid in 50% ACN, 5% TFA) and subjected to phosphoenrichment. Phosphopeptides were enriched using TiO_2_ beads (5020–75010; Capital HPLC Ltd, Broxburn, Scotland). Desalted samples were diluted to a volume of 500 µl using glycolic acid solution (1 M glycolic acid in 80% ACN, 5% TFA) and incubated for 5 min with 50 µL of titanium bead solution (500 µg/ml TiO_2_ beads in 1% TFA). Samples were centrifuged for 3 min at 1,500 rpm at room temperature (RT) and two thirds of the supernatant was transferred to fresh tubes. The pellets of TiO_2_ beads were resuspended in the remaining third of supernatant and pack into empty spin tips (TT2EMT; Glygen) by centrifugation. The supernatants collected in the previous step were run through the TiO_2_ loaded spin columns by centrifugation. TiO_2_ beads were sequentially washed by centrifugation with 100 µL of glycolic solution 2, ammonium acetate solution (100 mM ammonium acetate in 25% ACN) and twice with neutral solution (10% ACN). Phosphopeptides were eluted from the TiO_2_ beads by centrifugation with 4 times 50 µl of phosphopeptide elution solution (5% NH_4_OH). All centrifugations were done at 1,500 rpm at RT. Phosphopeptide solutions were frozen in dry ice for 15 min, dried in a speed vac and stored at -80 °C. Phosphopeptide pellets were re-suspended in 7 µl of reconstitution buffer (20 fmol/µl enolase in 3% ACN, 0.1% TFA) and 5 µl were injected into an LC-MS/MS platform. The LC system (Dionex UltiMate 3000 RSLC, Thermo-Fisher Scientific Inc) used mobile phases A (3 ACN: 0.1% FA) and B (100% ACN; 0.1% FA). Peptides were trapped in a µ-pre-column (160454; Thermo Fisher Scientific Inc) and separated in an analytical column (EASY-SPRAY RSLC C18 2 µM, 50 CM X 75 µM, #ES903, Thermo-Fisher Scientific Inc). The following parameters were used: 3% to 23% B gradient for 90 min and a flow rate of 0.25 µl/min. Samples were run in the Liquid chromatography–mass spectrometry/mass spectrometry LC-MS/MS system in a randomized manner by shuffling samples before loading. As they eluted from the nano-LC system, peptides were infused into the online connected Q-Exactive Plus system via an Easy Spray Source. The instrument operated a 2.1 s duty cycle consisting in the acquisition of a full scan survey spectra (375–1,500 m/z) with a 70,000 FWHM resolution was followed by, a data-dependent acquisition process in which the 15 most intense ions were selected for HCD (higher energy collisional dissociation) and MS/MS scanning (200–2,000 m/z) with a resolution of 17,500 FWHM. A 30 s dynamic exclusion period was enabled with an exclusion list with 10 ppm mass window. Overall duty cycle generated chromatographic peaks of approximately 30 s at the base, which allowed the construction of extracted ion chromatograms (XICs) with at least 10 data points. Peptide pellets for proteomics analysis were resuspended in reconstitution buffer (0.5 µg/µl) and 2 µl were injected into the LC-MS/MS platform. Samples were run in a 120 min gradient (3% to 23% B) and the rest of the LC-MS/MS parameters were kept as indicated for phosphoproteomics analysis.

### Phosphoenrichment, KSEA and GO enrichment analyses

Mascot Daemon 2.6.0 (http://www.matrixscience.com/mascot_support_v2_6.html*)* automated peptide identification from MS data. Mascot Distiller v2.7.1.0 generated Peak list files (MGFs) from RAW data. Mascot search engine (v2.5) matched data stored in MGF files to peptides. Searches were performed against the SwissProt Database (uniprot_sprot_2014_08.fasta) with a FDR of ~ 1% and the following parameters: 2 trypsin missed cleavages, mass tolerance of ± 10 ppm for the MS scans and ± 25 mmu for the MS/MS scans, carbamidomethyl Cys as a fixed modification, PyroGlu on N-terminal Gln, oxidation of Met and phosphorylation of Ser, Thr and Tyr as variable modifications. The in-house developed Pescal software was used for label-free peptide quantification, XICs for all the identified peptides across all samples were constructed with ± 7 ppm and ± 2 min mass and retention time windows, respectively. Peak areas from all XICs were calculated. Missed datapoints were given an intensity value equal to the minimal value obtained in the assessed sample divided by 10. Intensity values for each peptide were normalised to total sample intensity. For proteomics analysis, phosphorylation of Ser, Thr and Tyr was not considered in Mascot searches. Peptides were quantified with Pescal as previously indicated [25]. Protein amount was inferred using the quantitative data for all peptides identified in the same protein. Gene ontology (GO) enrichment analysis was carried using the inputs from the protein kinases obtained from phosphosites of the phosphoproteomic data, and Kolmogorov-Smirnoff test was performed to assess statistical significance.

### Survival analysis

OncoProExp: An interactive shiny web application for comprehensive cancer proteomics and phosphoproteomics analysis [49] was used to analyse phospho PRKACA expression in breast cancer patients from CPTAC pan-cancer cohort using LinkedOmics [48].

### Statistical analysis

Values are presented as mean ± SEM. Statistical significance was determined by using the proper analysis in Prism. For two samples, t-student were applied, for two or more samples with two conditions, we applied Two-Way ANOVA. In multiple comparison a Multiple-t-test was used. Differences were considered significant with a p value < 0.05.

## Supplementary Information

Below is the link to the electronic supplementary material.

Supplementary Material 1

## Data Availability

The RNA-seq datasets and phospho-proteomics data generated in this study have been deposited to the Gene Expression Omnibus (GEO) under accession number GSE314267, and GSE311784 and ProteomeXchange Consortium via the PRIDE partner repository under accession number PXD070990, respectively.
