# Supplementary material for: Tumour endothelial cell reprogramming orchestrates angiocrine signalling to drive chemoresistance in breast cancer
Source: Angiogenesis. 2026 Jun 22;29(3):48. doi: 10.1007/s10456-026-10063-7 (PMC13287203; doi:10.1007/s10456-026-10063-7)
Supplement: Supplementary file 1 — Supplementary Material 1 [file 10456_2026_10063_MOESM1_ESM.docx]

**Supplemental Information for**

**Tumour Endothelial Cell Reprogramming Orchestrates Angiocrine Signalling to Drive Chemoresistance in Breast Cancer**

Jesus Gomez-Escudero^1,2,6^, Eleni Maniati^1^, Julie Holdsworth^1^, Gordon Beattie^3,4^, Maruan Hijazi^1,2^, Matt Guelbert^5^, Samar Elorbany^1^, Pedro Cutillas^1^, Jun Wang^1^, Kairbaan Hodivala-Dilke^1,6^ , and Gabriela D’Amico^1,6^

^1^Barts Cancer Institute, Queen Mary University of London, Charterhouse Square, London, UK.

^2^Biochemistry and Molecular Biology Department, Salamanca University, Salamanca, Spain.

^3^CRUK City of London Centre Single Cell Genomics Facility, UCL Cancer Institute, University College London, London, UK.

^4^Bioinformatics Hub, UCL Cancer Institute, University College London, London, UK.

^5^Division of Cancer Biology, The Institute of Cancer Research, London, UK.

^6^Joint corresponding authors

**Keywords:** angiocrine, chemoresistance, breast cancer, NF-κB, Doxorubicin

This document contains:

- Fig.S1 to Fig.S11 (11 supplemental figures)
- Associated Legend Fig.S1 to Fig.S11 (11 supplemental figure legends)

******

***Fig. S1*.** **Prolonged Doxorubicin treatment promotes different response patterns in the MMTV-PyMT breast cancer model. a*.*** Plot shows tumour growth curves shown as tumour volume (mean ± SEM mm^3^) over time (days after treatment) for the same mice shown the shown in Fig.1b. *n* = 3 (Placebo, Pla), *n* = 6 (Responder to Dox; Rsp), *n* = 6 (Partial Responder to Dox; Prt) mice. Two-way ANOVA, Dunnett’s post-test. ***p<0.001. **b.** Bar plot shows percentage of days in which the tumour size from mice in (a) was found decreased (green) or increased (purple) compared to the previous day measurement. *n* = 6 (Rsp), *n* = 6 (Prt); ***p-value<0.001 (Two-way ANOVA, Sidak’s post-test). **c.** Plots depict change in tumour size in respect to previous measurement day (as fold change) generated with mice from 3 different experiments performed under same settings shown in Fig.1a. *n* = 3 (Pla), *n* = 16 (Rsp), *n* = 15 (Prt) mice. **d.** Bar plot shows percentage of days in which the tumour size from mice in (c) was found decreased (green) or increased (purple) compared to the previous measurement day. *n* = 16 (Rsp), *n* = 15 (Prt); **p-value<0.01 (Two-way ANOVA, Sidak’s post-test). **e.** Heatmap visualises significant regulated endothelial and non-endothelial/epithelial markers expression in bulk-RNAseq samples enriched in tumour endothelial cells (ECs) and in tumour cancer cells (Non-ECs) from placebo (Pla) and Dox-treated mice. *n* = 10 (ECs), *n* = 10 (Non-ECs). Bar plots show top pathway terms significantly deregulated in **f**. Rsp vs Pla and in **g.** Prt vs Pla tumour ECs. Colour gradient of bars represents adjusted p-values (Fisher’s exact test, Benjamini-Hochberg post-test for multiple hypotheses).

******

***Fig. S2.***  **Tumour endothelial cells exhibit distinct transcriptomic differences across prolonged Doxorubicin treatment response patterns.** **a.** Heatmap shows significant deregulated genes between Responder to Dox (Rsp) and Partial Responder to Dox (Prt) in non-endothelial cell (Non-EC) and endothelial cell (EC) samples isolated from MMTV-PyMT tumours from prolonged Doxorubicin treated mice. *n*= 5 non-EC Rsp, *n* = 3 Prt non-EC, *n*= 4 Rsp EC, and *n*= 3 Prt EC*.* **b.** Venn diagrams show significantly deregulated gene counts between Rsp and Prt samples in ECs and Non-ECs. **c.** Venn diagrams show significantly deregulated gene counts between Rsp and Prt related to Placebo (Pla) samples for ECs comparisons. Done with Venny app (https://bioinfogp.cnb.csic.es/tools/venny/index.htm).

******

***Fig. S3*. Representation of** **endothelial cell purity in the scRNA-seq analysis.** **a.** UMAP plots shows single endothelial cells isolated from MMTV-PyMT tumours from mice treated with Placebo or Doxorubicin (*n* = 3 pooled tumour samples per condition). Generated with ShinyCell app[91] based on normalized expression values. **b.** Bar plot represents the percentage of cells expressing each analysed gene from the UMAP plots (pct.express). Generated with R studio.

******

***Fig. S4.* Transcriptomic** **pathways changes in specific endothelial cell populations from the scRNA-seq analysis. a.** Gene Ontology terms analysis using positive marker genes from angiogenic clusters shown in Fig. 2f. **b.** Bar plot represents Hallmark pathway terms significantly deregulated using positive marker genes from cluster 8. Colour gradient of bars represents adjusted p-values (Fisher’s exact test). Generated with R studio. *n* = 3 pooled tumour samples per condition.

******

***Fig. S5*. Transcriptomic pathways changes in stress related endothelial cell populations in scRNA-seq analysis.** Bar plot shows top Hallmark pathway terms significantly deregulated using positive marker genes from clusters 0, 6 and 10. Colour gradient of bars represents adjusted p-values (Fisher’s exact test). Generated with R studio. *n* = 3 pooled tumour samples per condition.

***Fig. S6*. Angiocrine molecules expression in endothelial cell populations in scRNA-seq analysis.** Bubble plots show all angiocrine molecules expression within each endothelial cell cluster from prolonged Dox treated mice samples. Bubble size represents the ratio of cells in the cluster expressing a particular marker, and bubble colour represents marker expression levels. *n* = 3 pooled tumour samples per condition. Generated with ShinyCell app[91] based on normalized expression values.

***Fig. S7*. Angiocrine gene ontology terms analysis in distinct endothelial cell populations from sc-RNAseq analysis.** Bubble plot shows significant (-log Fisher’s exact test p value) cytokine, interleukin and chemokine gene ontology terms in each cluster of Responder to Dox (Rsp) and Partial Responder to Dox (Prt) MMTV-PyMT tumour-derived EC samples from prolonged Dox treated mice using the positive markers of each cluster. Generated with R studio. *n* = 3 pooled tumour samples per condition.

***Fig. S8*. scRNA-seq analysis of endothelial cells from acute Doxorubicin treated MMTV-PyMT tumours. a.** UMAP shows single endothelial cells isolated from Placebo (Pla) and acute Dox (Shrt) treated MMTV-PyMT tumour bearing mice. *n* = 3 pooled tumour samples per condition. **b.** Cell cluster abundance (in percentage) in each treated group. **c.**  Abundance of clusters (percentage) in each indicated EC cluster category, in each treated group (*n* = 3 pooled tumour samples per condition). **d.** Hallmark pathway terms analysis in Shrt versus Pla ECs. Fisher’s exact test p value. Generated with R studio. *n* = 3 pooled tumour samples per condition. **e.** Stress response and NF-kB markers, and **f**. Cytokine and chemokine-angiocrine characteristic molecules expression in angiocrine clusters 6 and 10 in Shrt versus Pla comparisons. Bubble size represents the percentage of cells in the cluster expressing a particular marker, and bubble colour represents marker expression levels. *n* = 3 pooled tumour samples per condition. Graphs generated with ShinyCell app[91] based on normalized expression values.

******

***Fig. S9*. Doxorubicin treatment *in vitro* changes angiocrine molecules protein expression in tumour endothelial cells*.* a.** Representative cell survival experiments images from tumour cancer cells derived from treatment naïve MMTV-PyMT tumour bearing mice (Non-ECs, two-dimenstional cultures, Crystal Violet cell survival assays). Tumour cancer cells were cultured in Optimem for 24 hours, then treated *in vitro* with Veh PBS control (vehicle, Veh) or 0.125μM Doxorubicin (Dox) for 72 hours. Quantification plot is provided as cell survival rate expressed as ratio against Veh control comparison. *n*= 4 biological repeats, 10 technical replicates in each and per condition. Mann-Whitney test. **** p-value<0.0001. Representative cytokine array blots of tumour endothelial cells isolated from MMTV-PyMT tumours and treated *in vitro* with **b.** vehicle PBS (Veh) or **c.** Doxorubicin (Dox) for 24 hours. *n* = 4 replicates from 2 membranes. **d.** Table shows the nomenclature for each selected (crop) analyte/cytokine shown in main Fig. 4. **e.** Bar plot shows expression of significantly deregulated angiocrine molecules from arrays in (b) assessed in the bulk-RNAseq EC samples analysis (Prt vs Rsp). Colour gradient of bars represents p-values (Fisher’s exact test with Benjamini-Hochberg post hoc test for multiple hypotheses). p-values numbers for each cytokine are included on within each bar.

******

***Fig. S10.* Conditional media from tumour endothelial cells treated with doxorubicin protects MMTV-pYMT-derived tumour cells from doxorubicin induced DNA damage *in vitro.* a.** Images from MMTV-PyMT-derived tumour cell 3D-spheroids pre-treated with conditioned media from endothelial cells that were treated with PBS control (vehicle, Veh) or Doxorubicin (0.125μm, Dox). Tumour spheroids were next treated with Dox at 0.125μM for 48 hours, fixed and stained for **a.** p-H2AX or **b.** Cleaved Caspase-3 (CC-3). Replicates from samples shown in Fig. 4d-e. *n* = spheroids (44) from 3 independent experiments. Scales, 100 μm.

******

***Fig. S11.* Conditional media from tumour endothelial cells treated with Doxorubicin *in vitro* protects breast tumour cells from Doxorubicin-driven DNA damage*.* a.** Representative cell survival experiments images, from tumour cancer cells primed for 24 hours with conditional media from tumour endothelial cells (ECs) that were exposed previously to PBS control (vehicle, Veh) or to 0.125μM Doxorubicin (Dox) for 24 hours *in vitro.* Next, the tumour cancer cells were treated with Veh or Dox (0.125μM) for additional 72 hours. Quantification plot is provided as cell survival rate expressed as ratio against Veh control comparisons. *n*= 4 biological repeats, 10 technical replicates in each. **** p-value <0.0001. *ns*, no significant (two-way ANOVA, Sidak’s post-test). **b.** Heatmap shows Ontology pathways related with DNA-damage and repair, obtained from phosphoproteomic datasets shown in Fig.4g. * represent significant regulated pathways (Kolgomorov-Smirnov test). *n* = 4 independent samples.
